# Loose Bowels

**Published:** 1859-07

**Authors:** 


### LOOSE BOWELS.

The first, most essential and most efficient step towards a cure in all cases, is that which instinct prompts; to wit: perfect quietude of body; next take nothing but rice parched like coffee; then boil, and eat in the usual way.

